# HIV and TB co-infection in the ART era: CD4 count distributions and TB case fatality in Cape Town

**DOI:** 10.1186/s12879-018-3256-9

**Published:** 2018-07-31

**Authors:** Richard Kaplan, Sabine Hermans, Judy Caldwell, Karen Jennings, Linda-Gail Bekker, Robin Wood

**Affiliations:** 1 The Desmond Tutu HIV Centre, Institute for Infectious Disease and Molecular Medicine, Faculty of Health Sciences, University of Cape Town, Anzio Road, Observatory, Cape Town, 7925 South Africa; 2 Department of Global Health, Academic Medical Center, University of Amsterdam, Amsterdam Institute for Global Health and Development, Amsterdam, the Netherlands; 3 Department of Internal Medicine, School of Medicine, Makerere University College of Health Sciences, Kampala, Uganda; 4 City Health, City of Cape Town, South Africa; 5 Department of Medicine, University of Cape Town, Cape Town, South Africa

**Keywords:** Tuberculosis, HIV, Antiretroviral therapy, Mortality, TB case fatality, CD4 count

## Abstract

**Background:**

In Cape Town, the roll-out of antiretroviral therapy (ART) has increased over the last decade with an estimated coverage of 63% of HIV- positive patients in 2013. The influence of ART on the characteristics of the population of HIV-positive patients presenting to the primary care TB programme is unknown. In this study, we examined trends in CD4 count distribution, ART usage and treatment outcomes among HIV-positive TB patients in Cape Town from 2009 to 2013.

**Methods:**

Data from the electronic TB register on all newly registered drug-sensitive TB patients ≥18 years were analyzed retrospectively. Descriptive statistics were used to compare baseline characteristics, the CD4 count distribution and TB treatment outcomes both by year of treatment and ART status at the start of TB treatment. Survival analyses were used to assess the change in mortality risk during TB treatment over time, stratified by ART status at start of TB treatment.

**Results:**

118,989 patients were treated over 5 years. HIV prevalence among TB patients decreased from 50.9% in 2009 to 49.0% in 2013. The absolute number of HIV-positive TB cases declined by 13.2% between 2010 and 2013. More patients entered the TB programme on ART in 2013 compared to 2009 (30.0% vs 9.9%). Among these, the CD4 count distribution showed a year by year shift to higher CD4 counts. In 2013, over 75% of ART-naïve TB patients still had a CD4 count < 350 cells/mm^3^. ART initiation among ART-naive patients increased from 37.0 to 77.7% and TB case fatality declined from 7.4 to 5.2% (*p* < 0.001). In multivariate analysis a decrease in TB mortality was most strongly associated with CD4 count (Adjusted HR 0.82 per increase of 50 cells/mm^3^, 95% CI: 0.81–0.83, *p* < 001) and the initiation of ART during TB treatment (Adjusted HR 0.39, 95% CI: 0.35–0.42, *p* < 0.001).

**Conclusion:**

Comprehensive changes in the ART and TB treatment programmes resulted in incremental increases in ART coverage for HIV-positive TB patients and a subsequent decrease in TB case fatality due to increased ART uptake in HIV-positive ART-naïve patients. However TB still remained a major presenting opportunistic infection with the majority of cases occurring at low CD4 counts.

**Electronic supplementary material:**

The online version of this article (10.1186/s12879-018-3256-9) contains supplementary material, which is available to authorized users.

## Background

In sub-Saharan Africa, the increase in the tuberculosis (TB) epidemic has been shown to strongly correlate with the increase in HIV-prevalence [1, 2]. This has been driven predominantly by a rise in HIV-positive TB cases among patients with severe immune suppression [2].

Public health interventions aimed at reducing TB incidence rates have included the WHO three I’s strategy of intensified case-finding, isoniazid preventive therapy and infection control as well as wide-scale antiretroviral therapy (ART) for HIV-positive patients [3]. In HIV-positive patients, ART has been shown to be strongly associated with a reduction in TB incidence rates [4]. However, previous studies of HIV/TB co-infected patients in sub-Saharan Africa have shown that most patients diagnosed with TB had low CD4 counts and were not receiving ART at the time of TB diagnosis [5–7].

In South Africa, the coverage of the ART rollout has increased substantially over the last decade [8]. In Cape Town with an HIV prevalence of 5.2% in 2013, the roll-out of ART in primary health care clinics was estimated to provide coverage of 63% of HIV-positive patients in that same year [9]. While the increase in coverage has been shown to coincide with a decline in the HIV-positive TB epidemic in the city, the influence of ART on the characteristics of the HIV-positive patients presenting with TB is unknown.

This study therefore aimed to determine the annual changes in HIV prevalence, CD4 count distribution, ART initiation and TB treatment outcomes among HIV-positive TB patients as well as the risk factors for TB case fatality in this cohort of patients receiving TB treatment in Cape Town from 2009 to 2013.

## Methods

### Setting and treatment

In Cape Town, TB treatment is provided in 101 primary care clinics by nursing staff with support from medical officers. During the period of the study, forty of these clinics also provided antiretroviral therapy with the remainder referring patients off- site for ART.

ART eligibility criteria and treatment regimens for HIV/TB co-infected patients changed twice during the period of the study. Prior to mid-2010, ART initiation was recommended for adult HIV-positive patients with a CD4 count <200copies/mm^3^ or WHO Stage 4 disease. The recommended first line treatment regimens were stavudine or zidovudine with lamivudine and efavirenz or nevirapine [10]. In 2010, updated South African ART guidelines extended the CD4 eligibility criteria to <350cells/mm^3^ and recommended a first line regimen of tenofovir, lamivudine and efavirenz and in August 2012, the guidelines were amended again to provide ART for all TB/HIV co-infected patients irrespective of the CD4 count [11, 12]. Second line ART was either zidovudine or tenofovir depending on prior exposure during first line treatment plus lamivudine and lopinavir/ritonavir. Due to rifampicin interactions with protease inhibitors, patients with TB on second line ART had the lopinavir/ritonavir boosted with extra ritonavir or the dose doubled slowly over a 3 week period [10, 11].

TB diagnosis and treatment guidelines also changed during the study period. Up to 2011, microbiological TB confirmation was determined through sputum smear microscopy or TB culture. [13] From August 2011, GeneXpert MTB/RIF was rolled out and by February 2013 it had completely replaced smear microscopy as the primary test for TB [14]. During the study period, new TB patients received treatment with a daily fixed dose combination tablet of rifampicin, isoniazid, pyrazinamide and ethambutol (RHZE) during the intensive phase of TB treatment followed by rifampicin and isoniazid (RH) in the continuation phase. Prior to 2013, retreatment TB patients received streptomycin 5 days a week for 2 months, rifampicin, isoniazid, pyrazinamide and ethambutol daily for 3 months, followed by rifampicin, isoniazid and ethambutol for a further 5 months. [13] In 2013, streptomycin was phased out and all patients with drug sensitive TB received RHZE in the intensive phase and RH in the continuation phase [14].

### Study design and population

This was a retrospective cohort analysis of newly registered drug sensitive TB patients ≥18 years. The data for the analysis was extracted from the electronic TB register (ETR.net) for Cape Town which is a database of case records of all patients who received TB treatment in primary care TB clinics in Cape Town. Included in the study were adult patients who started TB treatment in any of Cape Town’s primary health care TB treatment facilities from 1 January 2009 to 31 December 2013. To avoid duplication of cases, patients who transferred between primary care TB clinics for treatment were excluded. Although patients with drug resistant TB were not routinely entered into the ETR.net database, some patients in the database were subsequently noted to have drug resistant TB. These patients were also excluded from the analysis.

### Operational definitions

The TB definitions of TB treatment outcomes were according to the 2009 WHO TB Treatment Guidelines. [15] TB success was defined as the summation of cure and completion. TB case fatality was defined as death during TB treatment irrespective of cause of death. Patients who did not have a TB treatment outcome were recorded as “not evaluated”.

The HIV status of a patient was defined as positive if the patient was recorded to have a positive HIV test result at the beginning of TB treatment, or was recorded as receiving ART and/or co-trimoxazole prophylaxis and/or had a recorded CD4 cell count result at start of TB treatment. HIV-negative status was defined by a HIV-negative test result recorded in the database. All other patients were considered to be of unknown HIV status.

### Data analysis

Descriptive statistics were used to compare baseline characteristics, the CD4 count distribution and TB treatment outcomes both by year of treatment and ART status at the start of TB treatment. These were summarised as proportions for categorical variables and medians with inter-quartile ranges (IQRs) for continuous variables. Differences in HIV prevalence and median CD4 counts over time were tested using the chi-square test for trend and the Cuzick nonparametric test for trend respectively. Survival analyses were used to assess the change in mortality risk during TB treatment over time stratified by ART status at start of TB treatment (log-rank test for equality in survivor function). Censoring occurred at dates of death, default, transfer out, failure, cure or completion. Uni- and multivariable Cox proportional hazard models were used to determine risk factors for TB mortality in the HIV-positive patients on ART at start of TB treatment and not on ART at start of TB treatment. The proportionality assumption was assessed visually using –ln((−ln(survival)) plots. The analyses were performed using Stata IC 13.0 (StataCorp, USA).

### Ethics approval

The Cape Town City Health Directorate approved the use of an anonymised database of routinely collected TB data for this analysis. As the data used in this analysis were collected as part of the routine monitoring and evaluation of the South African National TB programme, patients were not requested to provide informed consent for the use of their data. Confidentiality was ensured through the removal of patient identifiers prior to the analysis. This study was approved by the University of Cape Town Research Ethics Committee.

## Results

### Cohort characteristics

Over the 5 year study period from 1 Jan 2009 to 31 Dec 2013, 149,360 TB patients were treated in 101 primary care clinics in Cape Town. Of these, 30,371 were excluded from this analysis as they were under the age of 18 (*n* = 22,468) or transferred care between the primary care TB clinics (*n* = 6746) or were found to have had drug resistant TB (*n* = 1157). Of the remaining 118,989 newly registered adult patients with drug sensitive TB, 60482 (50.8%) were HIV-positive and formed the cohort for this analysis (Additional file 1: Appendix 1). The remainder were either HIV-negative [55,377 (46.5%)] or had an unknown HIV status [3130 (2.6%)]. The HIV prevalence among TB patients decreased from 50.9% in 2009 to 49.0% in 2013. The absolute numbers of adult TB cases increased by 1.7% from 24,291 in 2009 to a peak of 24,697 in 2010 and then declined by 8.6% to 22,585 cases in 2013. This was largely due to a decline in HIV-positive TB cases. These first increased by 3.0% from 12,372 in 2009 to 12,749 in 2010 and then decreased by 13.2% to 11,060 in 2013. Over the same time period, HIV-negative TB cases increased by 5.6% from 10,673 in 2009 to 11,276 in 2010 with a marginal decrease of 54 cases (0.5%) between 2010 and 2013. The analysis of the 60,482 HIV-positive TB patients showed that 11,708 (19.4%) were on ART at the start of TB treatment while 48,749 (80.6%) were not on ART at the start of TB treatment.

### HIV-positive patients on ART

The patient characteristics of this cohort are shown by year in Table 1a. The proportion of patients on ART at the start of TB treatment increased over the 5 years from 9.9% in 2009 to 30.0% in 2013. The median age was 35 [interquartile range (IQR) 30–41 years] and 6799 (58.1%) were female. The proportion of extra-pulmonary TB cases decreased from 27.5% in 2009 to 16.8% in 2013. Over this time period, the median CD4 count at the start of TB treatment increased from 150 [IQR 74–242 cells/mm^3^] to 202 [IQR 91–348 cells/mm^3^]. There was a decline in retreatment cases particularly from 2012 to 2013 where the proportion of re-treatment cases decreased from 43.1 to 41.1%.

**Table 1:** Baseline characteristics by year of HIV-positive patients on ART (Section A) and not on ART (Section B) at the start of TB treatment

Start year	2009	2010	2011	2012	2013	Total
All HIV-positive patients	12,372	12,749	12,490	11,811	11,060	60,482
Section A.						
HIV-positive patients on ART *n* (%)	1221 (9.9)	1617 (12.7)	2235 (17.9)	3322 (28.1)	3313 (30.0)	11,708 (19.4)
Age, years, median [IQR]	35 [29–41]	34 [29–40]	36 [30–41]	35 [30–42]	36 [30–42]	35 [30–41]
Gender						
Female *n* (%)	746 (61.1)	980 (60.6)	1323 (59.2)	1870 (56.3)	1880 (56.7)	6799 (58.1)
Classification						
New TB *n* (%)	678 (55.5)	888 (54.9)	1249 (55.9)	1890 (56.9)	1953 (58.9)	6658 (56.9)
Retreatment TB *n* (%)	543 (44.5)	729 (45.1)	986 (44.1)	1432 (43.1)	1360 (41.1)	5050 (43.1)
Category						
PTB *n* (%)	885 (72.5)	1184 (73.2)	1725 (77.2)	2668 (80.3)	2758 (83.2)	9220 (78.7)
EPTB *n* (%)	336 (27.5)	433 (26.8)	510 (22.8)	654 (19.7)	555 (16.8)	2488 (21.3)
Median CD4	150 [74–242]	153 [75–254]	169 [80–290]	178 [80–315]	202 [91–348]	175 [81–305]
Section B.						
HIV-positive patients not on ART n (%)	11,142 (90.1)	11,130 (87.3)	10,255 (82.1)	8,481 (71.8)	7,741 (70.0)	48,749 (80.6)
Age, years, median [IQR]	34 [28-40]	34 [28-40]	34 [29-41]	35 [29-41]	35 [29-41]	34 [29-41]
Gender						
Female *n* (%)	5,839 (52.4)	5,973 (53.7)	5,367 (52.3)	4,313 (50.9)	3,892 (50.3)	25,384 (52.1)
Classification						
New TB *n* (%)	7,957 (71.4)	7,930 (71.2)	7,259 (70.8)	6,035 (71.2)	5,830 (75.3)	35,011 (71.8)
Retreatment TB *n* (%)	3,185 (28.6)	3,200 (28.8)	2,996 (29.2)	2,446 (28.8)	1,911 (24.7)	13,738 (28.2)
Category						
PTB *n* (%)	8,860 (79.5)	8,808 (79.1)	8,186 (79.8)	6,964 (82.1)	6,370 (82.3)	39,188 (80.4)
EPTB *n* (%)	2,282 (20.5)	2,322 (20.9)	2,069 (20.2)	1,517 (17.9)	1,371 (17.7)	9,561 (19.6)
Median CD4 count [IQR]	152 [69-281]	160 [72-289]	174 [78-308]	176 [74-319]	162 [67-313]	164 [72-300]

### HIV-positive patients not on ART

Table 1b shows the characteristics by year of the HIV-positive patients who were not on ART at the start of TB treatment. The median age was 34 [IQR 29–41 years] and 25,384 (52.1%) were female. In this cohort there was also a decline in the proportion of extra-pulmonary TB cases over the 5 years from 20.5% in 2009 to 17.7% in 2013 but this decline was less than in the patients on ART at the start of TB treatment. Re-treatment cases were unchanged between 2009 and 2012 but decreased from 28.8% in 2012 to 24.7% in 2013. The median CD4 count in this cohort was 152 [IQR 69–281 cells/mm^3^] in 2009, rising to 176 [IQR 74–319 cells/mm^3^] in 2012 but then decreasing to 162 [IQR 67–313 cells/mm^3^] in 2013.

When comparing the characteristics of the patients on ART with the patients who were not on ART at the start of TB treatment there were some differences between the two groups with patients on ART at start of TB treatment recording a higher proportion of retreatment TB cases compared to patients not on ART (43.1% vs 28.2%) and a higher proportion of extra-pulmonary TB (21.3% vs 19.6%). The majority of patients in both groups were female but more female patients were recorded on ART compared to not on ART (58.1% vs 52.1%) (Tables 1a and Table 1b).

### The CD4 count distribution and ART uptake

**Fig. 1:**
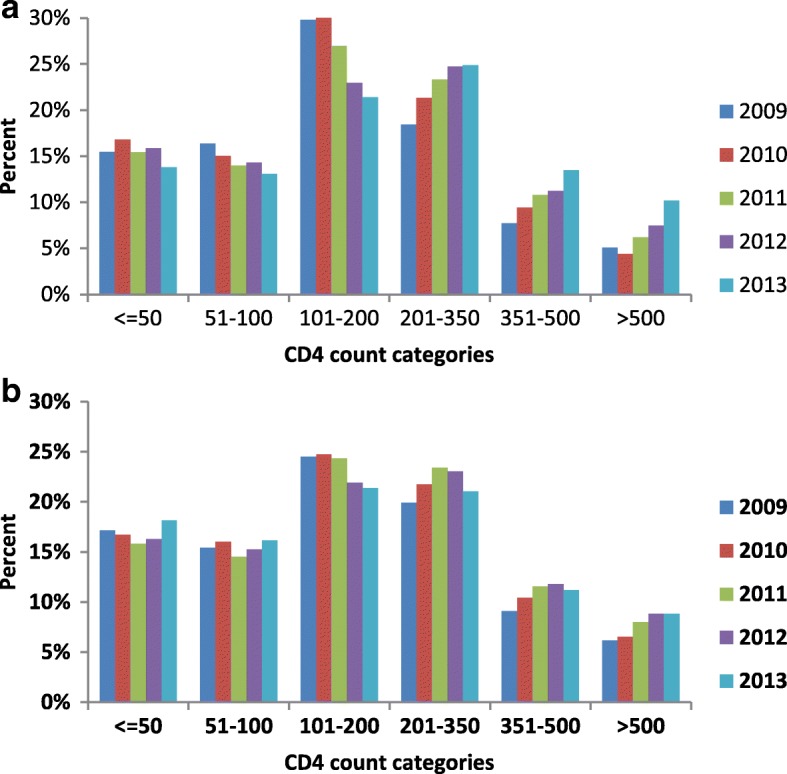
CD4 count distribution per year by ART status: **a** On ART at start of TB treatment . **b** Not on ART at start of TB treatment

Figure 1a shows the CD4 count distribution of the patients who were on ART at the start of TB treatment. The CD4 count distribution showed a small year by year shift from CD4 count categories less than 200 cells/mm^3^ to CD4 categories greater than 200 cells/mm^3^ with the greatest shift occurring in the CD4 category of 101–200 cells/mm^3^. In the patients who were not on ART at the start of TB treatment (Fig. 1b), there was also a shift in CD4 count distribution from the category 101–200 cells/mm^3^ to categories higher than 200 cells/mm^3^ but this change was far less pronounced.

ART uptake during TB treatment among those not on ART at the start of TB treatment increased substantially over the five year period from 37.0% in 2009 to 77.7% in 2013. However, when ART uptake was compared across CD4 categories, in 2013 despite guidelines that prescribed universal coverage for all HIV-positive TB patients, a greater proportion of patients with higher CD4 counts did not start ART: in the CD4 count category from 0 to 200 cells/mm^3^, 17.9% of patients did not start ART, in the category of 201–500 cells/mm^3^, 22.8% did not start ART and in category > 500 cells/mm^3^, 36.8% did not start ART. (data not shown).

**Fig. 2:**
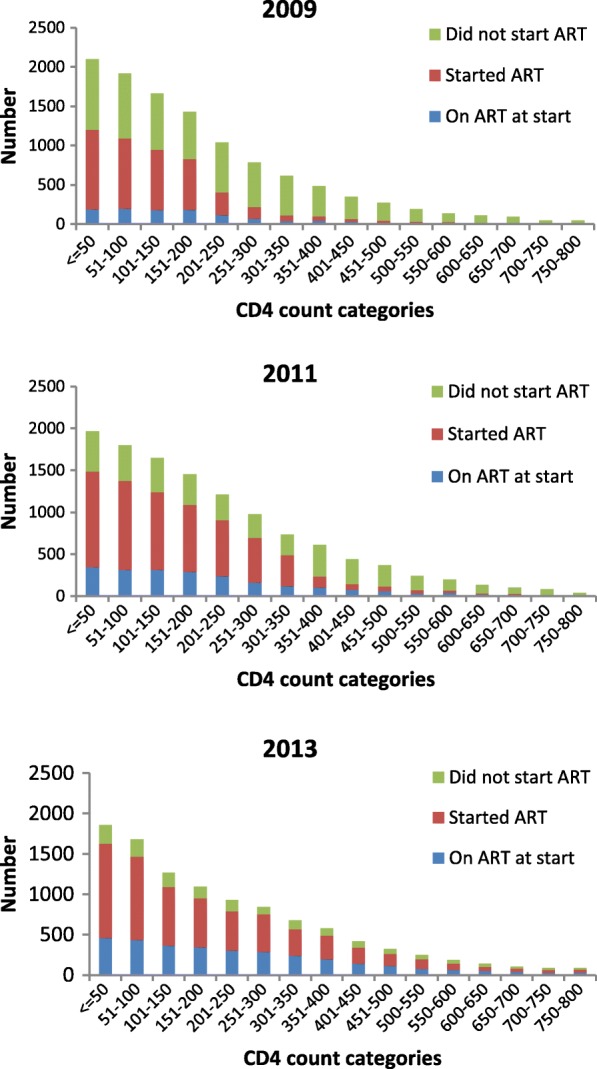
CD4 count distribution by ART status for the year 2009, 2011 and 2013

Figure 2 shows ART status by CD4 count categories per 50 cell/mm^3^ increase for the years 2009, 2011 and 2013. Over the five year period, total ART coverage of TB patients increased considerably both through the increase in numbers of patients presenting on ART as well as substantial increases in ART uptake during TB treatment for all CD4 count categories. Overall total ART coverage for HIV-positive TB patients during TB treatment increased from 45.0% in 2009 to 85.2% in 2013.

### TB treatment outcomes

There was an increase in TB treatment success rates over the 5 year period for both HIV positive TB patients who presented on ART (from 78.1 to 80.9%) and patients who were not on ART (76.7 to 79.6%) (Table 2). This was largely driven by a decrease in TB case fatality. For the patients who presented with TB while on ART, the TB case fatality decreased from 5.6% in 2009 to 4.7% in 2013 while for patients who were not on ART, the TB case fatality decreased from 7.4% in 2009 to 5.2% in 2013.

**Table 2:** TB treatment outcomes by year for HIV-positive patients by ART status at start of TB treatment

	Total	Success *n* (%)	Death *n* (%)	Default *n* (%)	Failure *n* (%)	Transfer Out *n* (%)	Not evaluated *n* (%)
HIV-positive patients (On ART at START)							
2009	1221	954 (78.1)	68 (5.6)	101 (8.3)	3 (0.3)	42 (3.4)	53 (4.3)
2010	1617	1280 (79.2)	102 (6.3)	138 (8.5)	8 (0.5)	45 (2.8)	44 (2.7)
2011	2235	1799 (80.5)	134 (6.0)	192 (8.6)	8 (0.4)	69 (3.1)	33 (1.5)
2012	3322	2651 (79.8)	178 (5.4)	309 (9.3)	19 (0.6)	122 (3.7)	43 (1.3)
2013	3313	2681 (80.9)	156 (4.7)	305 (9.2)	15 (0.5)	96 (2.9)	60 (1.8)
HIV-positive patients (Not on ART at START)							
2009	11,142	8546 (76.7)	826 (7.4)	1005 (9.0)	36 (0.3)	494 (4.4)	235 (2.1)
2010	11,130	8746 (78.6)	738 (6.6)	1009 (9.1)	40 (0.4)	449 (4.0)	148 (1.3)
2011	10,255	8135 (79.3)	619 (6.0)	977 (9.5)	24 (0.2)	363 (3.5)	137 (1.3)
2012	8481	6718 (79.2)	513 (6.1)	848 (10.0)	28 (0.3)	274 (3.2)	100 (1.2)
2013	7741	6162 (79.6)	403 (5.2)	731 (9.4)	23 (0.3)	265 (3.4)	157 (2.0)

### Survival analysis

**Fig. 3:**
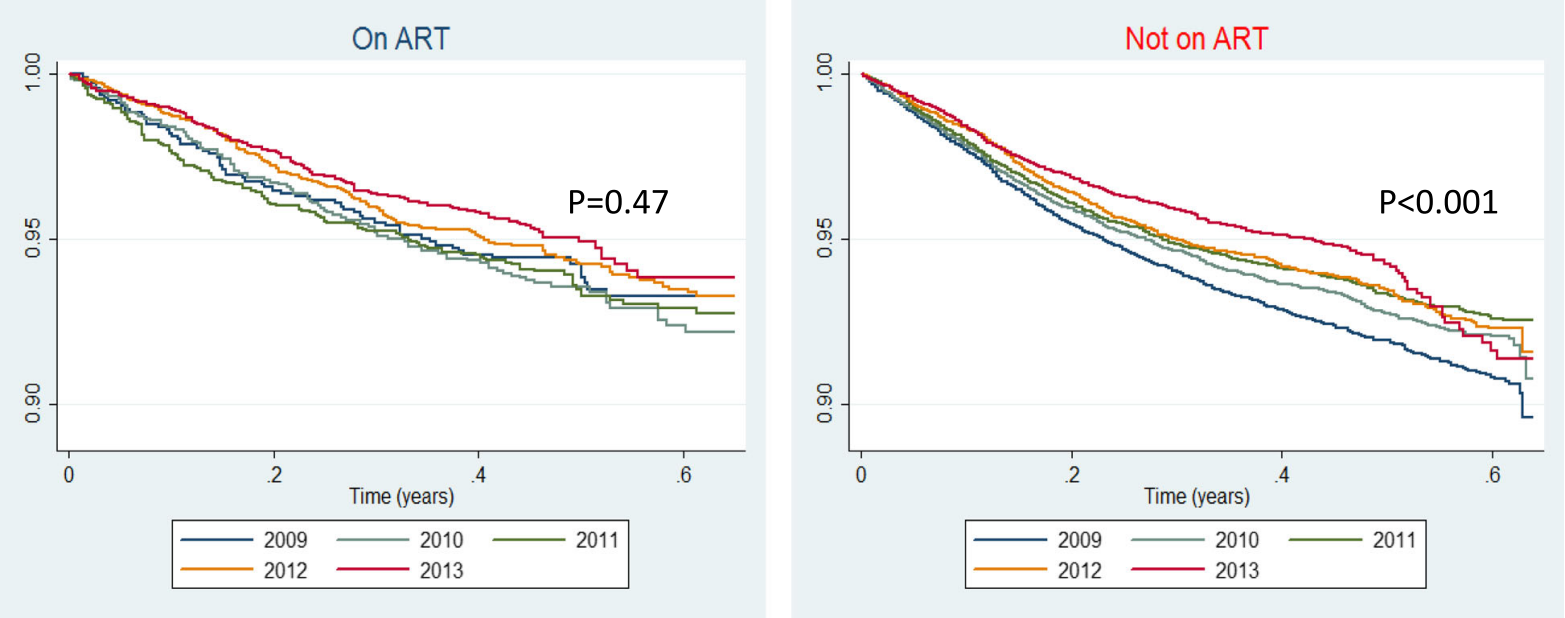
**a** Kaplan-Meier plot of survival by year for HIV-positive patients on ART at start of TB treatment (*p* = 0.47). **b** Kaplan-Meier plot of survival by year for HIV-positive patients not on ART at start of TB treatment (*p* < 001)

Figure 3 shows Kaplan–Meier plots of survival by year for the HIV-positive patients on ART (Fig. 3a) and not on ART at start of TB treatment (Fig. 3b). Survival by year for the HIV-positive patients on ART at the start of TB treatment was not significantly different over the 5 year period (*p* = 0.47) but for the patients not on ART at the start of TB treatment, the year on year improvement in survival was significant (*p* < 001).

### Risk factors for mortality during TB treatment for patient on ART and not on ART at the start of TB treatment

For patients on ART at the start of TB treatment, a multivariable analysis using Cox proportional hazard models showed a higher risk of TB mortality associated with increasing age (Adjusted hazard ratio (aHR) 1.28 per 10 years increase of age, 95% CI: 1.17–1.40, *p* < 0.001) and a lower risk of TB mortality associated with new TB (aHR 0.71, 95% CI: 0.61–0.84, *p* < 0.001) and increasing CD4 counts (aHR 0.87 per increase of 50 cells/mm^3^, 95% CI: 0.84–0.89, *p* < 0.001). TB mortality was not associated with calendar year of treatment, gender or type of TB. (Table 3).

**Table 3:** Univariable and multivariable analyses of factors associated with mortality for patients on ART at start of TB treatment

	PYRS	Deaths	Univariable		Multivariable	
Characteristic			HR (95% CI)	*P*-value	aHR (95% CI)	*P*-value
Calendar year						
2009	11,479	1400	1		1	
2010	11,646	1275	1.11 (0.81–1.51)	0.51	1.18 (0.85–1.63)	0.32
2011	11,350	1135	1.06 (0.79–1.42)	0.71	1.17 (0.86–1.59)	0.32
2012	11,095	1099	0.95 (0.72–1.26)	0.74	1.07 (0.80–1.44)	0.64
2013	10,010	922	0.90 (0.68–1.20)	0.47	1.05 (0.77–1.42)	0.76
Age (per 10 years increase)	5581	634	1.27 (1.17–1.38)	< 0.001	1.28 (1.17–1.40)	< 0.001
Female gender	3232	329	1		1	
Male gender	2349	305	1.28 (1.09–1.50)	0.002	1.02 (0.86–1.20)	0.84
Re-treatment TB	2606	340	1		1	
New TB	2975	294	0.72 (0.61–0.83)	< 0.001	0.71 (0.61–0.84)	< 0.001
PTB	4411	494	1		1	
EPTB	1170	140	1.06 (0.88–1.28)	0.56	1.05 (0.86–1.27)	0.43
CD4 (per increase of 50 cells/mm3)	5389	603	1 0.87 (0.84–0.89)	< 0.001	1 0.87 (0.84–0.89)	*P* < 0.001

*PYRS* person years, *HR* Hazard Ratio, *aHR* Adjusted Hazard Ratio, *CI* confidence interval, *TB* tuberculosis, *PTB* pulmonary tuberculosis, *EPTB* extra-pulmonary tuberculosis

Risk factors for TB mortality during TB treatment for HIV-positive patient not on ART at the start of TB treatment were increasing age, female gender, retreatment TB, and extra-pulmonary TB (Table 4). An increase in CD4 count was associated with a decrease in mortality (aHR 0.82 per increase of 50 cells/mm^3^, 95% CI: 0.81–0.83, *p* < 001) and the initiation of ART during TB treatment was most strongly associated with lower TB mortality (aHR 0.39, 95% CI: 0.35–0.42, *p* < 0.001).

**Table 4:** Univariable and multivariable analyses of factors associated with mortality for patients who were not on ART at start of TB treatment

	PYRS	Deaths	Univariable		Multivariable	
Characteristic			HR (95% CI)	*P*-value	aHR (95% CI)	*P*-value
Calendar year						
2009	5169	823	1	0.03	1	0.38
2010	5163	733	0.89 (0.81–0.98)	< 0.001	1.05 (0.94–1.18)	0.66
2011	4769	616	0.81 (0.73–0.90)	< 0.001	1.03 (0.91–1.16)	0.09
2012	3943	512	0.82 (0.73–0.91)	< 0.001	1.12 (0.98–1.27)	0.55
2013	3361	401	0.73 (0.65–0.83)		1.04 (0.91–1.21)	
Age (per 10 years increase)	22,405	3085	1.34 (1.30–1.39)	< 0.001	1.38 (1.32–1.44)	< 0.001
Female gender	11,662	1528	1	0.004	1	0.004
Male gender	10,743	1557	1.11 (1.03–1.19)		0.89 (0.82–0.96)	
Re-treatment TB	7142	1122	1		1	
New TB	15,263	1963	0.76 (0.71–0.82)	< 0.001	0.67 (0.62–0.73)	< 0.001
PTB	18,083	2391	1		1	
EPTB	4321	694	1.20 (1.11–1.31)	< 0.001	1.25 (1.14–1.38)	< 0.001
CD4 (per increase of 50 cells/mm3)	21,574	2807	0.85 (0.84–0.86)	< 0.001	0.82 (0.81–0.83)	< 0.001
Did not start ART	7037	1254	1		1	
Started ART	13,145	1301	0.56 (0.52–0.61)	< 0.001	0.39 (0.35–0.42)	< 0.001

*PYRS* person years, *HR* Hazard Ratio, *aHR* Adjusted Hazard Ratio, *CI* confidence interval, *TB* tuberculosis, *PTB* pulmonary tuberculosis, *EPTB* extra-pulmonary tuberculosis, *ART* antiretroviral therapy

## Discussion

Despite a great increase in the estimated ART coverage in Cape Town, the decrease in the HIV-positive adult TB case load was modest (13.2%). The effect of the ART treatment programme is apparent from the increase in numbers presenting on ART at the start of TB treatment and the upward shift in the CD4 count distribution of these patients. This is further supported by the decline in extra-pulmonary TB cases which is indicative of a less immunocompromised patient population.

However in 2013, 70% of HIV-positive TB patients were not on ART when presenting with TB, indicating that despite the roll-out of an extensive HIV treatment programme, TB still remained an important gateway to HIV care. The CD4 count distribution of these patients was largely unchanged with over 75% having a CD4 cell count less than 350 cells/mm^3^. For these patients, the multivariate analysis showed that ART uptake during TB treatment was most strongly associated with decreased TB mortality. Higher CD4 counts were also associated with decreased mortality but over the five year period, the rise in median CD4 count for patients who were not on ART at the start of TB treatment was modest indicating that the improved mortality was most likely driven by the considerable increase in ART uptake (37.0 to 77.7%). Total ART coverage for HIV-positive TB patients during TB treatment increased from 45.0% in 2009 to 85.2% in 2013.

This represents a substantial programmatic improvement in how TB/HIV coinfected patients have been managed in Cape Town. The decline in TB case fatality for TB patients who were not on ART from 7.4 to 5.2% compares very favourably with other treatment programmes. In a systematic review and meta-analysis on the impact of ART on mortality in HIV-positive patients on TB treatment, Odone et al. reported an estimated case fatality of 8–14% based on an analysis of 21 studies in Africa, Asia and the United Kingdom [16].

The low case fatality rate in Cape Town should however be interpreted with caution as the default rate of 9.4% in 2013 could partially represent unascertained mortality. The default rate however remained stable over the five year period for TB patients who were not on ART at the start of TB treatment. This confirms an overall absolute improvement in treatment outcomes as reflected in the higher combined cure and completion rate.

While the overall improvement in ART uptake from 2009 to 2013 was substantial, patients with higher CD4 counts in 2013 were less likely to start ART during TB treatment than those with lower CD4 counts. This may be partially due to the ART treatment guidelines which indicate that ART can be delayed for up to two months in patients with higher CD4 counts but may also be due to patient choice or clinicians’ practice [17]. While this is a small portion of the total population of TB cases, it does represent a missed opportunity to enrol patients on ART and could be detrimental to their long term prognosis particularly if ART is not started after the TB episode.

While this study shows improved ART uptake during TB treatment and improved TB treatment outcomes, the relatively small reduction in the HIV-positive TB case load does not provide support for ART as a highly effective method for curtailing the population TB case load. This is consistent with our recent evaluation of trends in TB notification rates during ART scale-up in Cape Town [9]. This is also supported by a number of modelling scenarios that indicate a limited effect of ART on TB incidence unless scale-up encompasses near universal coverage for all HIV-positive patients. [18–20].

The limitations of this study are the retrospective study design and the reliance on programmatic data. This is particularly apparent in the reported decline in retreatment cases from 2012 to 2013 which coincided with the phasing out of streptomycin in 2013 and may be due to an ascertainment error as retreatment cases were often identified by the inclusion of streptomycin in the TB treatment regimen [14]. A further limitation is that the data cut-off point for this study was end December 2013 and our findings therefore may not reflect the current TB prevalence and treatment outcomes in Cape Town.

## Conclusion

This study showed comprehensive changes in the ART and TB treatment programmes resulted in incremental increases in ART coverage for HIV-positive TB patients and a subsequent decrease in TB case fatality due to increased ART uptake in HIV-positive ART-naïve TB patients. However TB still remained a major presenting opportunistic infection with the majority of cases occurring at low CD4 counts.

## Additional file

Additional file 1: Appendix 1. Extract from the electronic TB register (ETR.net) of case records for all newly registered adult patients with drug sensitive TB who were treated in primary care TB clinics in Cape Town over the study period. (XLSX 12993 kb)

## Abbreviations

ART: Antiretroviral therapy
HIV: Human immunodeficiency virus
TB: Tuberculosis
